# Trigger-based adaptive proton therapy and changes in target coverage and cardiac dose in esophageal cancer

**DOI:** 10.1016/j.phro.2026.101104

**Published:** 2026-10-04

**Authors:** Vlad Badiu, Esther Kneepkens, Dominique Reijtenbagh, Maaike Berbée, Richard Canters

**Affiliations:** Department of Radiation Oncology (Maastro), GROW School for Oncology and Reproduction, Maastricht University Medical Centre+, Maastricht, Netherlands

**Keywords:** Adaptive proton therapy, Esophageal cancer, Dose accumulation, Intensity-modulated proton therapy, Cardiac sparing, Target coverage

## Abstract

**Background and purpose:**

Inter-fractional anatomical variations during proton therapy for esophageal cancer may compromise target coverage and increase dose to surrounding organs. Adaptive replanning can mitigate these effects, but its cumulative dose-based and clinical impact remains insufficiently characterized. We evaluated offline adaptive intensity-modulated proton therapy (IMPT) triggered by target underdose or cardiac overdose using deformable dose accumulation.

**Materials and methods:**

A retrospective cohort of 192 esophageal cancer patients treated with IMPT was analyzed. Eighty-five underwent adaptive replanning and 107 were treated non-adaptively. Dose accumulation was performed by assigning each fraction to the closest weekly repeat computed tomography (CT) scan and deformably mapping doses to the planning CT. Accumulated doses from adapted and non-adapted plans were compared for internal target volume (ITV) and gross tumor volume (GTV) coverage, target hotspots, heart and lung dose, and normal tissue complication probability (NTCP) for 2-year post-treatment mortality.

**Results:**

Compared with the non-adaptive treatment scenario, the adaptive scenario increased ITV D_98%_ by 1.9 percentage points (pp) and GTV D_98%_ by 0.8 pp. in the target underdose subgroup. In the cardiac overdose subgroup, mean heart dose decreased by 1.0 Gy and heart V_40Gy_ by 1.7 pp., with no increase in mean lung dose. NTCP for 2-year post-treatment mortality decreased by 1.5 pp. in this subgroup.

**Conclusions:**

Trigger-based adaptive IMPT improved cumulative target coverage and cardiac sparing without increasing lung dose. Target underdose and cardiac overdose triggers provided complementary benefits, supporting their use in structured adaptive replanning.

## Highlights

• Target underdose replanning increased internal target volume D_98%_ by 1.9 pp.
• Mean heart dose decreased from 12.4 to 11.4 Gy after adaptation.
• Heart-based adaptation reduced predicted 2-year mortality by 1.5 pp.
• Target triggers improved coverage, while cardiac triggers reduced heart dose.

## Graphical abstract

**Figure:**
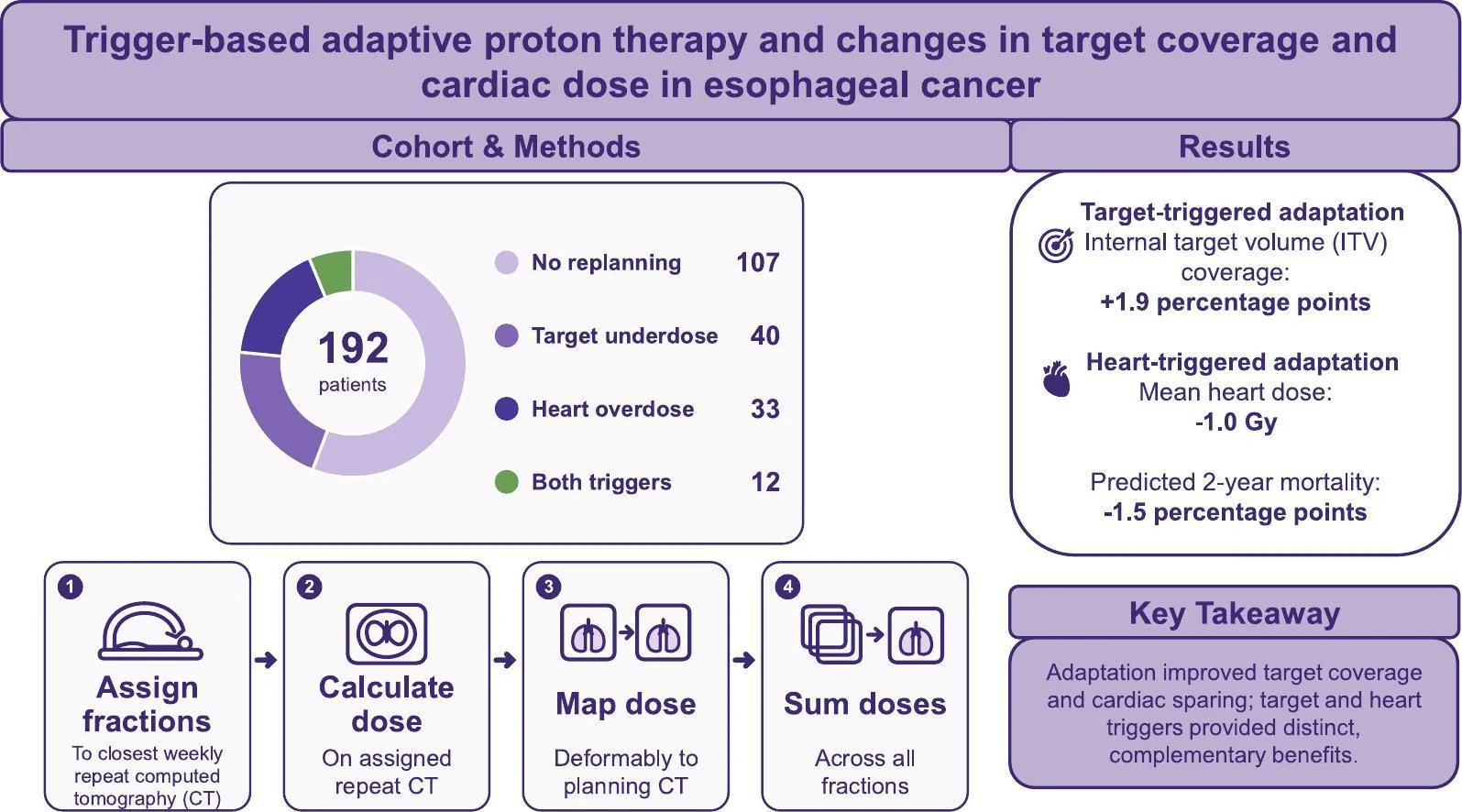

## 1 Introduction

Multimodality treatment including chemoradiotherapy plays a crucial role in the treatment of esophageal cancer, substantially improving overall survival [1], [2]. However, this approach is also associated with a risk of radiation-associated adverse effects, including pulmonary and cardiac complications [3], [4], [5], [6]. Studies have demonstrated an association between cardiac irradiation and overall survival following radiotherapy for esophageal cancer, emphasizing the importance of minimizing heart dose whenever possible [7], [8], [9].

Intensity modulated proton therapy (IMPT) exploits the sharp dose gradients of proton beams to achieve highly conformal dose distributions, thereby reducing dose to surrounding normal tissues. This can result in improved sparing of thoracic organs compared with conventional photon radiotherapy, particularly for esophageal cancer [10], [11]. However, proton therapy remains highly sensitive to anatomical changes during treatment, particularly for thoracic indications. Esophageal cancer patients frequently exhibit inter-fractional anatomical changes such as tumor regression, tumor displacement, tissue deformation, and weight loss, all of which can lead to substantially altered dose distributions and may compromise both target coverage and organ-of-interest (OOI) sparing if not adequately accounted for [12], [13].

Adaptive radiotherapy has been proposed as a strategy to mitigate the impact of inter-fractional anatomical changes through treatment replanning. Traditionally, adaptation in esophageal cancer has been primarily driven by concerns regarding target underdose [12], [14]. More recently, heart dose has emerged as a clinically relevant adaptation trigger, motivated by its demonstrated impact on survival and its incorporation into national proton therapy indication protocols [9], [15]. However, the dose-based and clinical consequences of heart-based adaptation, particularly in relation to target coverage and cumulative delivered dose, require additional investigation.

An added challenge lies in the evaluation of adaptive treatments. While plan quality is typically assessed on individual repeated computed tomography (CT) scans (reCTs), such assessments may not capture the cumulative effect of anatomical changes over time. Dose accumulation may offer a more comprehensive evaluation of delivered dose by accounting for fraction-to-fraction variability, but its application in adaptive proton therapy for esophageal cancer has been limited by practical constraints related to image registration accuracy, workflow complexity, and computational demands [16], [17], [18]. In this study, routinely acquired weekly reCTs and a previously validated dose accumulation workflow made retrospective comparison of cumulative doses feasible, while registration uncertainty remained a consideration [16].

The aim of this retrospective study was to evaluate the impact of offline, trigger-based adaptive IMPT on target coverage, OOI dose, and predicted 2-year post-treatment mortality risk in esophageal cancer patients using deformable dose accumulation. Specifically, we compared accumulated dose distributions representing adaptive and non-adaptive workflows, investigated the influence of different adaptation triggers (e.g. target underdose versus heart overdose), and assessed the clinical relevance of these dose differences using normal tissue complication probability (NTCP) for 2-year post-treatment mortality.

## 2 Materials and methods

### 2.1 Patient data

This retrospective evaluation included 192 consecutive esophageal cancer patients treated with IMPT at our institution between 2019 and 2025. No additional clinical selection criteria were applied. Detailed patient and tumor characteristics are provided in Supplementary Table S1. Patients received either neoadjuvant chemoradiotherapy with 23 × 1.8 Gy or definitive chemoradiotherapy with 28 × 1.8 Gy [19], [20], [21]. Each patient had an average-breathing planning CT (pCT) and between one and six reCTs, all routinely acquired once a week during treatment on the same imaging system, with a mean of four reCTs per patient (median: 4; interquartile range: 4–4). The average-breathing CTs were reconstructed from eight respiratory phases of each four-dimensional CT acquisition, covering the full inhale–exhale cycle. Of the 192 patients, 85 underwent adaptive replanning during treatment, whereas none of the remaining 107 patients met the clinically applicable adaptation criteria. Clinical adaptation was triggered by (i) target underdose, defined as internal target volume (ITV) D_98%_ below 94% of prescription dose in the voxel-wise minimum (VWmin) plan, (ii) an increase in mean heart dose (MHD) of ≥1.5 Gy relative to the dose on the pCT, or (iii) a combination of both [22]. The heart-based adaptation trigger was introduced clinically in June 2022. Before that, adaptation was based on target underdose only. Of the 85 patients who underwent adaptation, 33 underwent replanning due to heart overdose, 40 due to target underdose (27 prior to June 2022 and 13 thereafter), and 12 due to target underdose and heart overdose detected on different reCTs during treatment. All patient data were pseudonymized prior to analysis under institutional review board approval (code P0707), which determined that informed consent was not required for this retrospective evaluation.

### 2.2 Treatment planning

Target delineation followed published esophageal cancer recommendations [21]. The ITV was generated from target delineations on the eight respiratory-phase images of the planning 4DCT to account for respiratory motion; no breath-hold or repainting strategy was applied. Treatments were delivered using the S250i Hyperscan proton therapy system (Mevion Medical Systems, USA) [23]. Initial IMPT plans were created in RayStation (RaySearch Laboratories, Sweden) using pencil beam scanning with a standardized three-field configuration (posterior-oblique and anterior-oriented beams), robustly optimized on the average CT with ±5 mm setup and ± 3% range uncertainties. Adapted plans retained the initial beam arrangement and robustness settings, while the dose values and weights of the optimization objectives were adjusted as needed during replanning. Optimization prioritized target coverage while minimizing heart and lung dose. A 3-mm dose grid and a square spot pattern were used, with automatic energy-layer spacing (factor 1) and automatic spot spacing (factor 0.4 for small targets and 0.5 for large targets). Dose calculations were performed using the RayStation Monte Carlo dose engine (version 5.7) with a 1% statistical uncertainty setting. All reported proton dose values assume a constant relative biological effectiveness (RBE) of 1.1 and are expressed in Gy(RBE), in accordance with the International Commission on Radiation Units and Measurements (ICRU) Report 78 [24]. The pCT and reCTs were rigidly registered in RayStation 12 A using the gray-level-based frame-of-reference registration tool with emphasis on bony anatomy. Structures were copied from the 50% exhale breathing phase to the average-breathing pCT and subsequently transferred from the pCT to the reCTs using RayStation's hybrid intensity and structure-based deformable image registration algorithm (ANACONDA). Post-transfer to the reCTs, the structures were visually reviewed and manually corrected where required to match the anatomy on the reCT. Each patient began treatment with an initial plan, with up to three subsequent replans introduced as clinically indicated.

### 2.3 Dose accumulation

Dose accumulation was performed in RayStation 12 A using an in-house developed Python 3.11 script. Deformable dose mapping from each reCT to the pCT was performed using the RayStation deformable image registration with controlling regions of interest (ROIs) for the target, heart, and lungs, following the previously validated approach described by Taasti et al. [16]. For each patient, treatment fractions were assigned to the reCT closest in time to account for anatomical variations during the treatment course. Dose distributions were calculated on the assigned reCTs, deformably mapped to the pCT, and subsequently summed across all fractions and plans to generate the nominal accumulated dose distribution.

To account for residual geometric and range uncertainties, a robust evaluation was performed, following the Dutch practical robustness evaluation approach [22], [25], [26]. Residual setup uncertainties of 2 mm were simulated by applying isocenter shifts in all translational directions, combined with ±3% range uncertainty. Fourteen setup uncertainties were combined with two range uncertainties (−3% and + 3%), resulting in 28 perturbed scenarios. For each scenario, the dose distribution was computed, mapped to the pCT, and accumulated across all delivered fractions and plans. VWmin and VWmax dose distributions were then derived from the set of accumulated scenario doses, providing conservative estimates of underdose and overdose across scenarios, in accordance with the methodology described by Taasti et al. [16].

Two accumulation strategies were implemented. In the Multiple Plan Accumulation (MPA) approach, all clinically delivered plans were included: the initial plan and any subsequent adapted plans. This represents the adaptive workflow in which replans were introduced when triggered by target underdose or OOI overdose. In the Single Plan Accumulation (SPA) approach, only the initial plan was used across all fractions, regardless of whether replanning occurred clinically. SPA therefore represents a non-adaptive workflow, retrospectively simulated for adapted patients and clinically delivered for non-adapted patients. The effect of adaptation was evaluated within the adapted patients by comparing SPA and MPA; non-adapted patients were evaluated with SPA only. Comparisons between adapted and non-adapted patients were descriptive. For both approaches, nominal, VWmin, and VWmax summed doses were generated on the pCT used in subsequent dose/volume and NTCP analyses.

### 2.4 Evaluation and statistical analysis

Dose and volume metrics were evaluated on the pCT after deformable mapping and accumulation of the dose distributions; therefore, transferred reCT structures were not used for final dose-volume histogram (DVH) extraction.

From the accumulated dose distributions, target coverage was evaluated using ITV D_98%_ and gross tumor volume (GTV) D_98%_ from the VWmin dose, and ITV D_max_ from the VWmax dose. For OOIs, the nominal accumulated dose was used to determine MHD, mean lung dose (MLD), and heart V_40Gy_. These metrics were selected to reflect clinically relevant target coverage and hotspots, as well as cardiac and pulmonary exposure used in treatment evaluation and adaptation.

Patients who underwent adaptation for a combination of both criteria at any time during treatment were excluded from subgroup comparative analysis. For adapted patients, accumulated target and OOI dose metrics were compared between the SPA and MPA using paired *t*-tests. Given the sample size of the paired comparisons (*n* = 85), the sampling distribution of the mean paired difference was assumed to be approximately normal. Statistical significance was defined as *p* < 0.05. In addition, MHD was used to calculate the NTCP for 2-year post-treatment mortality according to the Dutch national indication protocol for proton therapy [15], and NTCP values were likewise compared between approaches.

## 3 Results

Target coverage metrics for all 192 patients, expressed as ITV and GTV D_98%_ as well as ITV D_max_, are presented in Fig. 1 and Table 1. Mean ITV D_98%_ was 97.5% in non-adapted patients evaluated with SPA. In adapted patients, ITV D_98%_ was 95.8% evaluated with SPA and increased to 97.1% with MPA, which accounted for all clinically delivered plans. Paired comparisons in the adapted patients demonstrated a statistically significant improvement in ITV D_98%_ with MPA (*p* = 0.028). A similar trend was observed for GTV coverage. Mean GTV D_98%_ increased from 98.9% to 99.7% in adapted patients when comparing SPA with MPA (*p* < 0.001). In patients adapted for target underdose, ITV D_98%_ increased from 95.0% to 96.9% and GTV D_98%_ from 98.9% to 99.7% with MPA. In addition, ITV D_max_ decreased from 108.4% to 107.4% in adapted patients (*p* < 0.001), indicating a reduction in target hotspots when accounting for adaptation.

**Fig. 1:**
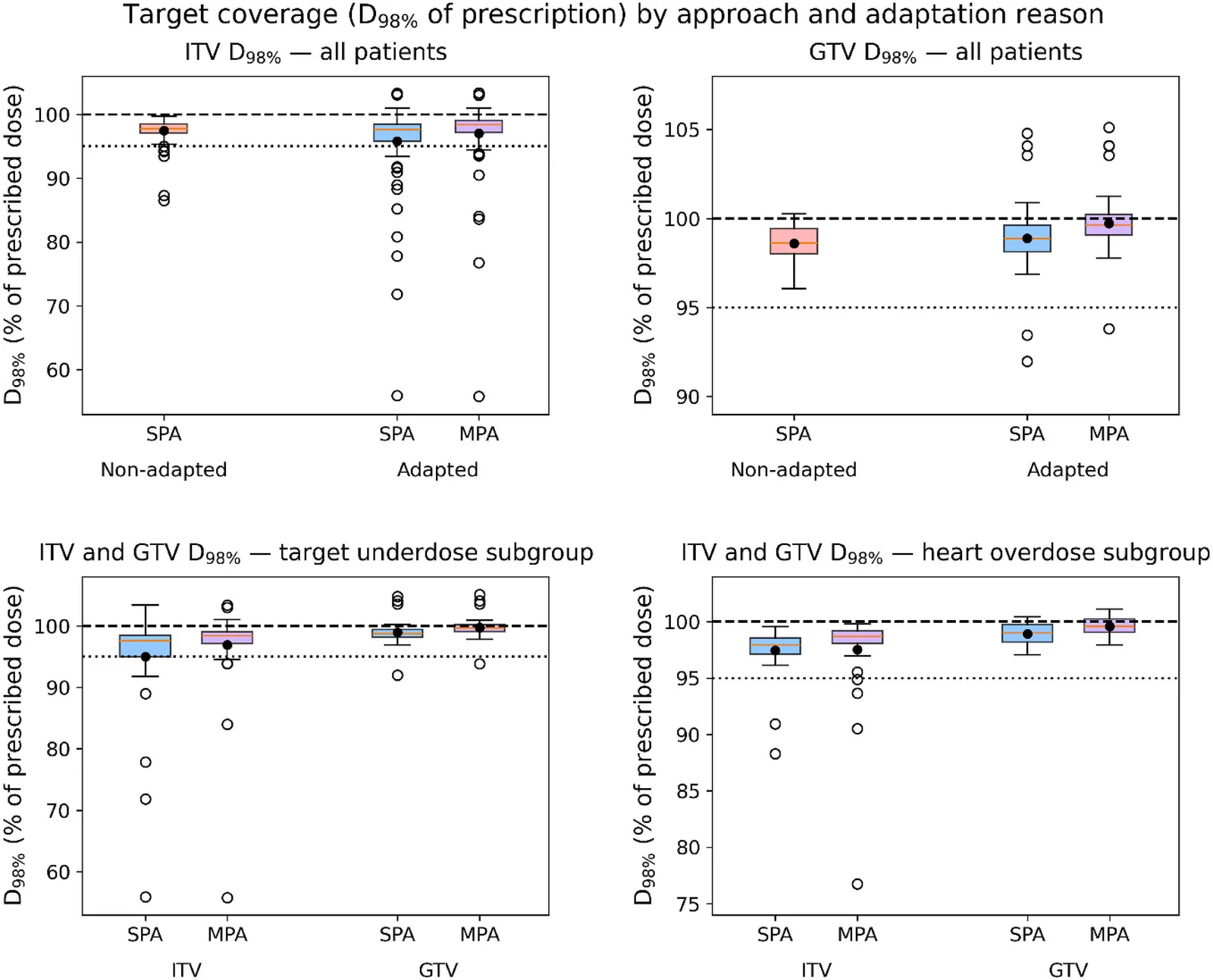
Boxplots of ITV and GTV D_98%_ coverage. Top row: all patients (SPA non-adapted, SPA adapted, MPA adapted). Bottom row: adapted patients only, split by adaptation reason. Left: patients adapted due to target underdose, showing ITV and GTV coverage (SPA vs MPA). Right: patients adapted due to heart overdose, showing ITV and GTV coverage (SPA vs MPA). Horizontal dashed and dotted lines indicate 100% and 95% of the prescription dose, respectively. Boxes represent the interquartile range with median lines; whiskers extend to 1.5 × IQR, open circles indicate outliers, and black circles indicate means. Blue and lilac represent SPA and MPA, respectively. (For interpretation of the references to colour in this figure legend, the reader is referred to the web version of this article.)

**Table 1:** Mean ± standard deviation of ITV and GTV D_98%_ and ITV D_max_ (%) (as % of prescription dose) for all patients and adapted patients (SPA vs MPA).

Metric	All patients	Target underdose patients (*n* = 40)	Heart overdose patients (*n* = 33)	*p*-value (SPA vs MPA, adapted patients)
ITV D_98%_ – non-adapted SPA	97.5 ± 1.9	–	–	
ITV D_98%_ – adapted SPA	95.8 ± 6.4	95.0 ± 8.5	97.5 ± 2.3	0.028
ITV D_98%_ – adapted MPA	97.1 ± 5.9	96.9 ± 7.3	97.5 ± 4.2	
GTV D_98%_ – non-adapted SPA	98.6 ± 1.0	–	–	
GTV D_98%_ – adapted SPA	98.9 ± 1.6	98.9 ± 2.0	98.9 ± 0.9	<0.001
GTV D_98%_ – adapted MPA	99.7 ± 1.4	99.7 ± 1.8	99.6 ± 0.7	
ITV D_max_ – non-adapted SPA	107.8 ± 1.4	–	–	
ITV D_max_ – adapted SPA	108.4 ± 1.9	108.4 ± 2.3	108.5 ± 1.5	<0.001
ITV D_max_ – adapted MPA	107.4 ± 1.7	107.5 ± 2.0	107.3 ± 1.0	

The OOI dose metrics for heart (MHD and V_40Gy_) and lung (MLD) are presented in Fig. 2 and Table 2. In the adapted patients, MHD decreased from 12.0 Gy to 11.6 Gy when comparing SPA with MPA (*p* = 0.002). Similarly, heart V_40Gy_ decreased from 7.9% to 7.0% (*p* < 0.001). In contrast, MLD remained unchanged between approaches (5.9 Gy in both cases, *p* = 0.969).

**Fig. 2:**
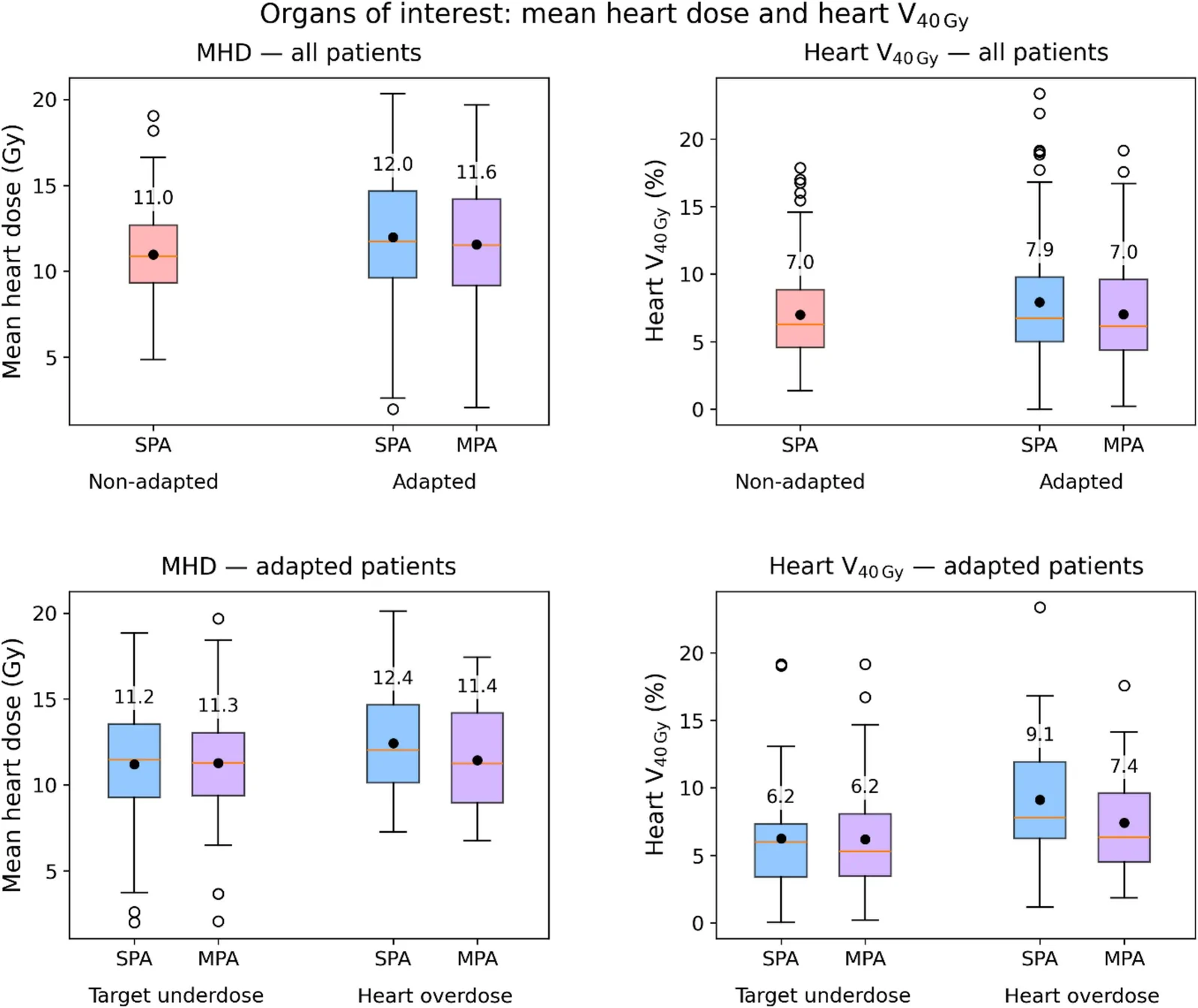
Boxplots of mean heart dose (MHD) and heart V_40Gy_. Top row: all patients (SPA non-adapted, SPA adapted, MPA adapted). Bottom row: adapted patients only, split by adaptation reason. Left: MHD for patients adapted due to target underdose and heart overdose, shown separately for SPA and MPA. Right: heart V_40Gy_ for patients adapted due to target underdose and heart overdose, shown separately for SPA and MPA. Boxes represent the interquartile range with median lines; whiskers extend to 1.5 × IQR, open circles indicate outliers, and black circles indicate means. Numerical labels indicate group mean values for mean heart dose (Gy) and heart V_40Gy_ (%).

**Table 2:** Mean ± standard deviation of heart dose (Gy), heart V_40Gy_ (%), and mean lung dose (Gy) for all patients and adapted patients (SPA vs MPA).

Metric	All patients	Target underdose patients (n = 40)	Heart overdose patients (n = 33)	p-value (SPA vs MPA, adapted patients)
MHD – non-adapted SPA	11.0 ± 2.8	–	–	
MHD – adapted SPA	12.0 ± 3.8	11.2 ± 3.9	12.4 ± 3.4	0.002
MHD – adapted MPA	11.6 ± 3.4	11.3 ± 3.9	11.4 ± 3.0	
Heart V_40Gy_ – non-adapted SPA	7.0 ± 3.5	–	–	
Heart V_40Gy_ – adapted SPA	7.9 ± 5.2	6.2 ± 4.3	9.1 ± 4.9	<0.001
Heart V_40Gy_ – adapted MPA	7.0 ± 4.5	6.2 ± 4.3	7.4 ± 3.8	
MLD – non-adapted SPA	5.5 ± 2.4	–	–	
MLD – adapted SPA	5.9 ± 3.4	5.0 ± 2.6	6.9 ± 3.8	0.969
MLD – adapted MPA	5.9 ± 3.3	5.2 ± 2.6	6.7 ± 3.8	

The NTCP values for 2-year post-treatment mortality are presented in Fig. 3 and Table 3. In the adapted patients, the mean NTCP decreased from 30.9% with SPA to 30.3% with MPA (*p* < 0.001). In patients adapted for heart overdose, MHD decreased from 12.4 to 11.4 Gy, heart V_40Gy_ from 9.1% to 7.4%, and NTCP from 33.9% to 32.4%.

**Fig. 3:**
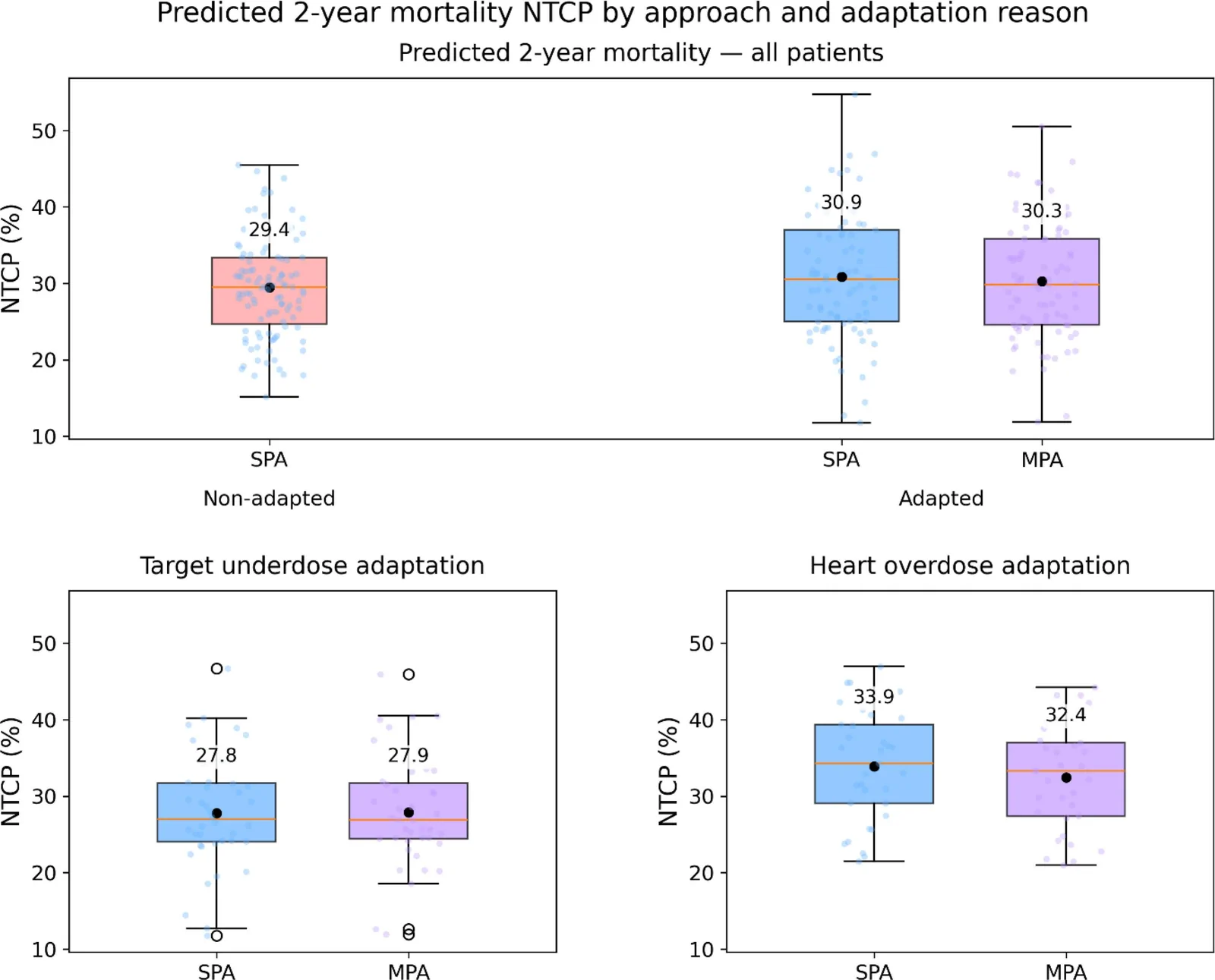
Boxplots of NTCP with data points for the individual patients overlaid. Top: all patients (SPA non-adapted, SPA adapted, MPA adapted). Bottom row: adapted patients only, split by adaptation reason. Left: patients adapted due to target underdose, showing SPA vs MPA. Right: patients adapted due to heart overdose, showing SPA vs MPA. Boxes represent the interquartile range with median lines; whiskers extend to 1.5 × IQR, open circles indicate outliers, and black circles indicate means. Faint colored points represent individual patient values. Numerical labels indicate group mean predicted 2-year post-treatment mortality risk (%).

**Table 3:** Normal tissue complication probability ± standard deviation for 2-year post-treatment mortality in all patients and adapted patients (SPA vs MPA).

NTCP (%)	Mean – all patients (%)	Target underdose patients (%) n = 40	Heart overdose patients (%) n = 33	P-value (SPA vs MPA, adapted patients)
Non-adapted SPA	29.4 ± 6.5	–	–	
Adapted SPA	30.9 ± 8.2	27.8 ± 7.5	33.9 ± 7.9	<0.001
Adapted MPA	30.3 ± 7.6	27.9 ± 7.3	32.4 ± 6.8	

## 4 Discussion

This retrospective study evaluated the impact of adaptive IMPT on cumulative target coverage and OOI dose in esophageal cancer using deformable dose accumulation, and assessed the influence of target underdose and heart overdose adaptation triggers. Adaptive replanning improved cumulative target coverage and reduced cardiac dose, while MLD remained unchanged. Target-triggered adaptation primarily improved coverage, whereas heart-triggered adaptation reduced cardiac exposure and the estimated NTCP for 2-year post-treatment mortality. These findings indicate that both triggers provide complementary information during treatment. Because adaptation was clinically triggered according to the clinical adaptation criteria, adapted and non-adapted patients may differ in baseline characteristics, anatomical instability, and susceptibility to dose deterioration; observed differences between groups should therefore be interpreted pragmatically.

Retrospective dose accumulation with SPA showed that 7 of 107 non-adapted patients (6.5%) failed to meet the ITV coverage goal, although none had met the clinical adaptation criteria on individual reCTs during treatment. Among the adapted patients, 17 of 85 (20.0%) failed the coverage goal with SPA, compared with 12 of 85 (14.1%) with MPA. All 12 patients had met the ITV coverage goal in their initial clinical plans. Persistent undercoverage in the accumulated dose may reflect dose delivered before replanning or pronounced anatomical changes during treatment, including tumor regression and associated changes in proton beam range, which have previously been characterized in the same patient cohort [27]. Compared with SPA, MPA increased ITV D_98%_ in 69 of 85 adapted patients (81.2%). In the remaining 16 patients, ITV D_98%_ was numerically lower with MPA than with SPA, although 9 maintained clinically acceptable coverage with MPA. These findings indicate that adaptive proton therapy safeguarded target coverage for most patients, while a subset showed limited benefit, likely reflecting substantial anatomical change or spontaneous recovery of coverage on later anatomy. This pattern is consistent with prior radiotherapy studies showing that inter-fractional anatomical changes in esophageal cancer can compromise target coverage and that adaptive strategies can improve robustness [12], [13], [14], [28].

The overall adaptation rate in this cohort was 44.3% (85/192), which is broadly consistent with previous esophageal radiotherapy adaptation studies, although direct comparison is limited by differences in technique, imaging frequency, setup strategy, and adaptation criteria [29], [30], [31]. The inclusion of cardiac overdose alongside target underdose likely contributed to the observed rate.

Dose accumulation enables evaluation of the cumulative delivered dose over the treatment course, which is particularly relevant in proton therapy because anatomical variability and range uncertainties may alter dose distributions. By integrating fraction-to-fraction changes, dose accumulation provides a more realistic representation of target coverage and healthy-tissue exposure than single-timepoint evaluation. However, its accuracy depends on deformable image registration, which is uncertain in the presence of tumor regression, weight loss, and tissue deformation. Validation of the applied framework showed median absolute deformable image registration-related differences of 0.31 percentage points for ITV D_98%_, 0.62 Gy for MHD, and 0.09 Gy for MLD in esophageal cancer patients, although larger local discrepancies occurred below the diaphragm [16]. The assumed scenario-wise systematic uncertainties may not fully reflect daily stochastic variations, and differences between reCT acquisition and the exact treatment position may introduce additional geometric uncertainty. The 0.62 Gy median absolute difference in MHD associated with deformable registration is larger than the 0.4 Gy mean MHD decrease across all the adapted patients and represents a considerable proportion of the 1.0 Gy decrease in the heart overdose subgroup. This validation measure does not directly estimate uncertainty in the paired SPA–MPA differences, but warrants caution when interpreting small MHD changes. Because dose accumulation was performed on average-breathing CTs, intra-fraction respiratory motion and spot-interplay effects were not explicitly modeled and may contribute additional uncertainty to the accumulated dose estimates.

Heart-based adaptation did not negatively affect ITV coverage, indicating that cardiac-triggered replanning can be introduced without sacrificing target dose. Although target-based triggers drove the largest coverage gains, adapted patients showed lower average D_98%_ values and larger variability overall, consistent with larger anatomical instability in this group.

ITV D_max_ of adapted patients was on average 1.0 percentage point higher when evaluated with SPA, indicating that failing to adapt not only reduced coverage but also increased the risk of hotspots within the target. Although this difference was modest, it reflects increased dose heterogeneity that may arise when anatomical changes are not accounted for during treatment.

Adaptation did not meaningfully change the MLD, supporting the absence of a lung-dose trade-off. The higher average lung doses observed in patients adapted for cardiac overdose compared with those adapted for target underdose may reflect differences in baseline anatomy and treatment characteristics rather than an effect of adaptation itself. This finding supports that adaptation can be implemented without compromising lung sparing.

Cardiac dose reductions were largest after heart-triggered adaptation, whereas target-triggered adaptation produced minimal cardiac dose changes, underscoring the need for explicit cardiac monitoring and trigger criteria. This aligns with literature linking cardiac dose to overall survival and with efforts to integrate heart-dose guidance and NTCP-based selection into proton therapy workflows [9], [15], [32].

These results highlight the value of MHD as a clinically meaningful adaptation trigger. Patients adapted for excessive MHD achieved consistent reductions in both MHD and V_40Gy_, supporting integration of cardiac dose monitoring into adaptive workflows. Importantly, adaptation was implemented in response to evolving anatomical changes rather than suboptimal initial planning. In the target-triggered subgroup, 10 patients (25%) failed the ITV D_98%_ criterion when evaluated with SPA, compared with 5 patients (12.5%) after MPA, demonstrating that adaptation mitigated coverage deterioration in a substantial proportion of these cases.

For patients with persistent undercoverage, online adaptation could allow more timely correction of evolving anatomy [33]. Whether this approach could also allow for safe margin reduction requires further investigation. While a 1.5 Gy MHD increase was used to initiate cardiac adaptation, earlier intervention or refined thresholds may further limit cumulative cardiac exposure, particularly in the context of progressive anatomical change.

The potential clinical relevance of these dose reductions was explored using the NTCP analysis. In patients adapted for heart overdose, MPA reduced the predicted 2-year mortality from 33.9% to 32.4%, amounting to a reduction of approximately 1.5 percentage points. This reduction is of a similar order of magnitude to the 2% NTCP difference used in the Dutch national indication protocol to guide proton-versus-photon modality selection [15]. However, its clinical significance in the context of adaptive replanning remains uncertain, particularly given the limited literature on this topic [6], [34].

In contrast, target underdose patients showed stable NTCP values between SPA and MPA, consistent with their adaptation primarily benefiting target coverage rather than cardiac sparing. The lower accumulated MHD observed in non-adapted patients should not be interpreted as an effect of non-adaptation, as the cohorts differed by adaptation indication and patients with unfavorable cardiac dose changes were more likely to undergo heart-triggered replanning. Our findings support the ability of trigger-based adaptation to mitigate increases in cardiac dose during treatment, but do not directly establish preservation of the initially predicted proton-versus-photon therapeutic advantage. The NTCP results should nevertheless be interpreted as estimates of potential clinical impact, since the model used in the Dutch indication protocol was derived from photon therapy data using planned dose parameters, whereas this study applied it to accumulated proton dose distributions. Similarly, target coverage criteria developed for robust plan evaluation may not be directly interchangeable with accumulated dose evaluation, and future work should investigate whether separate criteria are needed.

In conclusion, robust planning combined with systematic replanning represents an effective strategy to optimize target coverage and cardiac sparing in routine esophageal proton therapy. Structured, trigger-based adaptation may help preserve the therapeutic advantage of proton therapy throughout treatment, even in anatomically dynamic thoracic patients.

## Declaration of generative AI in scientific writing

During the preparation of this work, the authors used ChatGPT (OpenAI, San Francisco, California) to enhance the clarity of the grammar, spelling and general comprehensibility of the text. After using this tool, the authors reviewed and edited the content as needed and take full responsibility for the content of the published article.

## CRediT authorship contribution statement

**Vlad Badiu:** Writing – review & editing, Writing – original draft, Visualization, Validation, Project administration, Methodology, Investigation, Formal analysis, Data curation, Conceptualization. **Esther Kneepkens:** Writing – review & editing, Visualization, Validation, Supervision, Project administration, Methodology, Investigation, Conceptualization. **Dominique Reijtenbagh:** Writing – review & editing, Visualization, Methodology, Investigation, Conceptualization. **Maaike Berbée:** Writing – review & editing, Visualization, Supervision, Methodology, Investigation. **Richard Canters:** Writing – review & editing, Visualization, Validation, Supervision, Project administration, Methodology, Investigation, Formal analysis, Data curation.

## Ethics declaration

The authors have NOT obtained informed consent from participants or their legal representatives. Maastro's institutional review board (approval P0707) determined that informed consent was not required for this retrospective evaluation. All patient data were pseudonymized prior to analysis, and no identifiable patient information is reported.

This study was performed in compliance with relevant laws, regulatory frameworks and guidelines where the research took place.

This study was approved by the Maastro institutional review board.

(Approval No. P0707)

## Declaration of competing interest

The authors declare that they have no known competing financial interests or personal relationships that could have appeared to influence the work reported in this paper.

## Appendix A Supplementary data

Supplementary material: Supplementary Table S1. Patient and treatment characteristics
