# Supplementary material for: Trigger-based adaptive proton therapy and changes in target coverage and cardiac dose in esophageal cancer
Source: Phys Imaging Radiat Oncol. 2026 Oct 4;41:101104. doi: 10.1016/j.phro.2026.101104 (PMC13672842; doi:10.1016/j.phro.2026.101104)
Supplement: Supplementary file 1 [file mmc1.pdf]

Supplementary Table S1. Patient and treatment characteristics of the 192 patients.

Note: Age is given in years; fractions denotes the number of radiotherapy fractions. AC, adenocarcinoma; SCC, squamous cell carcinoma; nCRT, neoadjuvant chemoradiotherapy; dCRT, definitive chemoradiotherapy. Clinical T and N refer to primary tumor and regional lymph node stage, respectively. All patients were treated with curative intent.

Patient#	Fractions	Age	Sex	Histology	Tumor location	Clinical T stage	Clinical N stage	Tumor stage	Treatment schedule	Number of replans
**1**	23	74	female	AC	distal	2	0	IIB	nCRT	1
**2**	23	55	male	AC	distal	3	0	III	nCRT	1
**3**	23	71	male	AC	distal	3	2	IVA	nCRT	1
**4**	23	64	male	SCC	mid	3	1	III	nCRT	1
**5**	23	73	male	AC	distal	2	0	IIB	nCRT	2
**6**	28	74	male	AC	distal	2	0	IIB	dCRT	2
**7**	23	45	male	AC	distal	3	1	III	nCRT	1
**8**	28	78	female	AC	distal	2	1	III	dCRT	1
**9**	23	63	male	AC	distal	3	1	III	nCRT	2
**10**	28	87	male	AC	distal	3	1	III	dCRT	1
**11**	23	60	male	AC	distal	3	0	III	nCRT	1
**12**	28	81	female	AC	distal	3	1	III	dCRT	3
**13**	28	80	female	AC	distal	2	1	III	dCRT	1
**14**	23	69	male	AC	distal	3	1	III	nCRT	1
**15**	23	54	male	AC	distal	3	1	III	nCRT	1
**16**	23	53	female	AC	distal	3	1	III	nCRT	1
**17**	23	63	male	AC	distal	3	1	III	nCRT	2
**18**	23	76	female	AC	distal	3	0	III	nCRT	1
**19**	23	72	male	AC	distal	3	0	III	nCRT	1
**20**	23	64	male	AC	distal	3	2	IVA	nCRT	2
**21**	23	64	male	AC	distal	3	1	III	nCRT	1
**22**	23	58	male	AC	distal	3	3	IVA	nCRT	1
**23**	23	62	male	AC	distal	3	2	IVA	nCRT	1
**24**	28	78	female	AC	distal	3	2	IVA	dCRT	1
**25**	28	84	male	AC	distal	3	0	III	dCRT	1
**26**	23	66	male	AC	distal	3	2	IVA	nCRT	2
**27**	23	71	male	AC	distal	3	0	III	nCRT	1
**28**	23	63	female	SCC	distal	2	0	II	nCRT	2
**29**	23	67	male	AC	distal	2	1	III	nCRT	1
**30**	23	75	male	AC	distal	2	1	III	nCRT	2
**31**	23	76	male	AC	distal	NA	0	NA	nCRT	1

Supplementary Table S1 (continued)

Patient#	Fractions	Age	Sex	Histology	Tumor location	Clinical T stage	Clinical N stage	Tumor stage	Treatment schedule	Number of replans
**32**	23	65	male	AC	distal	3	0	III	nCRT	1
**33**	28	78	male	AC	distal	3	2	IVA	dCRT	2
**34**	23	66	male	AC	distal	3	0	III	nCRT	1
**35**	23	77	male	AC	distal	3	1	III	nCRT	1
**36**	28	76	male	SCC	mid	3	1	III	dCRT	1
**37**	23	69	male	AC	distal	3	0	III	nCRT	1
**38**	23	58	male	AC	distal	1	2	IVA	nCRT	1
**39**	23	58	male	AC	distal	3	2	IVA	nCRT	1
**40**	23	73	male	AC	distal	3	1	III	nCRT	1
**41**	28	82	male	AC	distal	3	0	III	dCRT	2
**42**	23	56	female	SCC	distal	2	2	III	nCRT	1
**43**	23	72	male	SCC	mid	3	1	III	nCRT	3
**44**	23	73	male	SCC	distal	3	1	III	nCRT	1
**45**	28	79	male	AC	distal	2	2	IVA	dCRT	1
**46**	23	75	male	AC	distal	3	1	III	nCRT	1
**47**	23	72	male	AC	distal	3	0	III	nCRT	2
**48**	28	71	female	SCC	distal	3	0	II	nCRT	1
**49**	23	47	male	AC	distal	3	1	III	nCRT	1
**50**	23	60	female	SCC	distal	3	1	III	nCRT	2
**51**	28	62	male	AC	distal	2	0	IIB	dCRT	1
**52**	23	71	male	AC	distal	3	2	IVA	nCRT	2
**53**	23	39	male	AC	distal	3	0	III	nCRT	1
**54**	23	81	male	AC	distal	3	1	III	nCRT	1
**55**	28	66	male	AC	distal	2	2	IVA	nCRT	3
**56**	23	60	male	AC	distal	2	0	IIB	nCRT	2
**57**	23	55	female	large cell	mid	3	0	NA	nCRT	1
**58**	23	66	male	AC	distal	3	0	III	nCRT	1
**59**	23	60	male	AC	distal	2	1	III	nCRT	1
**60**	23	67	male	AC	distal	3	2	IVA	nCRT	1
**61**	23	74	male	AC	mid	2	1	III	nCRT	1
**62**	23	72	male	AC	distal	3	0	III	nCRT	1
**63**	23	75	male	AC	distal	3	1	III	nCRT	1
**64**	28	71	male	AC	distal	3	1	III	dCRT	1
**65**	23	58	male	AC	distal	3	3	IVA	nCRT	1
**66**	28	75	male	SCC	distal	3	0	II	dCRT	1
**67**	23	61	male	AC	distal	3	1	III	nCRT	1
**68**	23	71	female	AC	distal	2	1	III	nCRT	2

Supplementary Table S1 (continued)

Patient#	Fractions	Age	Sex	Histology	Tumor location	Clinical T stage	Clinical N stage	Tumor stage	Treatment schedule	Number of replans
**69**	23	74	female	AC	mid	3	1	III	nCRT	1
**70**	23	55	male	AC	distal	2	1	III	nCRT	1
**71**	23	70	male	AC	distal	2	1	III	nCRT	1
**72**	23	83	male	AC	distal	2	1	III	nCRT	1
**73**	28	74	male	AC	distal	3	1	III	nCRT	1
**74**	23	78	male	AC	distal	2	0	IIB	nCRT	2
**75**	23	69	female	SCC	mid	3	1	III	nCRT	1
**76**	23	71	male	AC	distal	3	2	IVA	nCRT	1
**77**	23	63	male	AC	distal	3	1	III	nCRT	2
**78**	23	71	male	AC	distal	3	1	III	nCRT	1
**79**	23	67	male	AC	distal	2	1	III	nCRT	1
**80**	23	66	female	AC	distal	2	1	III	nCRT	1
**81**	23	74	male	AC	distal	2	0	IIB	nCRT	2
**82**	23	74	male	AC	distal	2	0	IIB	nCRT	1
**83**	23	74	female	SCC	mid	2	0	II	nCRT	1
**84**	23	73	male	AC	distal	3	0	III	nCRT	1
**85**	28	78	female	AC	distal	2	0	IIB	dCRT	1
**86**	23	71	male	AC	distal	2	1	III	nCRT	0
**87**	23	71	female	SCC	mid	3	1	III	nCRT	0
**88**	23	64	female	SCC	distal	2	0	II	nCRT	0
**89**	23	65	male	AC	distal	3	0	III	nCRT	0
**90**	23	73	female	AC	distal	2	0	IIB	nCRT	0
**91**	23	74	male	AC	distal	2	1	III	nCRT	0
**92**	23	65	female	SCC	mid	3	0	II	nCRT	0
**93**	23	57	male	AC	distal	2	1	III	nCRT	0
**94**	28	58	male	AC	distal	3	0	III	dCRT	0
**95**	23	64	male	AC	distal	3	0	III	nCRT	0
**96**	23	74	male	AC	distal	3	2	IVA	nCRT	0
**97**	23	72	male	AC	distal	3	0	III	nCRT	0
**98**	23	79	male	AC	distal	2	2	IVA	nCRT	0
**99**	23	56	male	AC	distal	2	2	IVA	nCRT	0
**100**	23	66	male	AC	distal	3	0	III	nCRT	0
**101**	23	71	male	AC	distal	3	1	III	nCRT	0
**102**	23	66	male	AC	distal	3	0	III	nCRT	0
**103**	23	64	male	AC	distal	3	0	III	nCRT	0
**104**	23	64	male	AC	distal	3	2	IVA	nCRT	0
**105**	23	64	male	SCC	mid	3	0	II	nCRT	0

Supplementary Table S1 (continued)

Patient#	Fractions	Age	Sex	Histology	Tumor location	Clinical T stage	Clinical N stage	Tumor stage	Treatment schedule	Number of replans
**106**	23	66	male	SCC	distal	3	1	III	nCRT	0
**107**	23	68	female	AC	distal	3	0	III	nCRT	0
**108**	28	72	male	AC	distal	3	0	III	dCRT	0
**109**	28	72	female	SCC	distal	2	0	II	dCRT	0
**110**	23	68	female	SCC	mid	3	1	III	nCRT	0
**111**	23	53	male	AC	distal	3	2	IVA	nCRT	0
**112**	23	57	male	AC	distal	2	0	IIB	nCRT	0
**113**	28	85	female	SCC	distal	3	0	II	dCRT	0
**114**	23	63	female	AC	distal	2	0	IIB	nCRT	0
**115**	23	79	male	AC	distal	3	1	III	nCRT	0
**116**	28	84	male	SCC	mid	3	1	III	dCRT	0
**117**	23	81	male	AC	distal	2	0	IIB	nCRT	0
**118**	23	73	male	AC	distal	3	2	IVA	nCRT	0
**119**	23	75	male	AC	distal	3	1	III	nCRT	0
**120**	23	69	male	AC	mid	2	0	IIB	nCRT	0
**121**	28	81	male	AC	distal	3	0	III	dCRT	0
**122**	23	58	male	AC	distal	3	2	IVA	nCRT	0
**123**	23	68	male	AC	distal	3	1	III	nCRT	0
**124**	28	82	male	SCC	distal	3	0	II	dCRT	0
**125**	28	59	male	AC	distal	3	2	IVA	nCRT	0
**126**	28	56	male	AC	upper	2	0	IIB	dCRT	0
**127**	23	61	male	AC	distal	2	1	III	nCRT	0
**128**	23	55	male	AC	distal	2	1	III	nCRT	0
**129**	23	70	male	AC	distal	3	1	III	nCRT	0
**130**	23	61	male	AC	distal	3	1	III	nCRT	0
**131**	23	61	male	AC	distal	3	1	III	nCRT	0
**132**	23	64	male	AC	distal	3	1	III	nCRT	0
**133**	28	75	male	AC	distal	2	0	IIB	dCRT	0
**134**	23	67	female	AC	distal	2	0	IIB	nCRT	0
**135**	23	40	male	AC	distal	2	1	III	nCRT	0
**136**	23	69	male	AC	mid	3	2	IVA	nCRT	0
**137**	23	64	male	AC	distal	3	1	III	nCRT	0
**138**	23	60	male	SCC	distal	3	2	III	nCRT	0
**139**	23	57	male	SCC	distal	2	2	III	nCRT	0
**140**	23	57	male	AC	distal	3	0	III	nCRT	0
**141**	23	66	male	AC	distal	3	1	III	nCRT	0
**142**	23	62	male	AC	distal	3	2	IVA	nCRT	0

Supplementary Table S1 (continued)

Patient#	Fractions	Age	Sex	Histology	Tumor location	Clinical T stage	Clinical N stage	Tumor stage	Treatment schedule	Number of replans
**143**	23	73	female	AC	distal	2	0	IIB	nCRT	0
**144**	23	72	male	AC	distal	3	0	III	nCRT	0
**145**	23	65	male	AC	distal	3	0	III	nCRT	0
**146**	23	68	female	SCC	mid	2	1	II	nCRT	0
**147**	23	66	male	AC	distal	3	1	III	nCRT	0
**148**	23	60	male	AC	distal	3	0	III	nCRT	0
**149**	23	78	female	AC	distal	3	0	III	nCRT	0
**150**	28	56	male	AC	mid	3	2	IVA	dCRT	0
**151**	23	63	male	AC	distal	3	3	IVA	nCRT	0
**152**	23	59	male	AC	distal	3	2	IVA	nCRT	0
**153**	23	69	male	AC	distal	2	2	IVA	nCRT	0
**154**	23	63	male	AC	distal	2	1	III	nCRT	0
**155**	23	52	male	AC	distal	3	1	III	nCRT	0
**156**	28	67	male	SCC	distal	2	1	II	dCRT	0
**157**	23	72	male	AC	distal	3	2	IVA	nCRT	0
**158**	28	63	male	AC	distal	3	2	IVA	dCRT	0
**159**	23	40	male	AC	distal	3	0	III	nCRT	0
**160**	23	72	male	AC	distal	3	2	IVA	nCRT	0
**161**	23	76	female	SCC	mid	3	0	II	nCRT	0
**162**	23	58	male	AC	distal	3	1	III	nCRT	0
**163**	23	65	male	AC	distal	3	2	IVA	nCRT	0
**164**	23	71	male	AC	distal	3	0	III	nCRT	0
**165**	23	61	female	SCC	distal	3	1	III	nCRT	0
**166**	23	70	male	AC	distal	3	2	IVA	nCRT	0
**167**	23	67	female	AC	distal	3	2	IVA	nCRT	0
**168**	23	70	male	SCC	distal	3	1	III	nCRT	0
**169**	23	80	male	AC	distal	2	0	IIB	nCRT	0
**170**	23	68	male	AC	distal	2	0	IIB	nCRT	0
**171**	23	73	male	SCC	mid	3	0	II	dCRT	0
**172**	23	66	female	AC	distal	3	1	III	nCRT	0
**173**	23	71	male	AC	distal	3	1	III	nCRT	0
**174**	23	72	male	AC	distal	2	1	III	nCRT	0
**175**	23	45	male	AC	distal	3	2	IVA	nCRT	0
**176**	23	62	female	AC	distal	3	0	III	nCRT	0
**177**	23	59	male	AC	distal	3	1	III	nCRT	0
**178**	23	58	male	AC	distal	3	2	IVA	nCRT	0
**179**	23	75	female	AC	distal	3	0	III	nCRT	0

Supplementary Table S1 (continued)

Patient#	Fractions	Age	Sex	Histology	Tumor location	Clinical T stage	Clinical N stage	Tumor stage	Treatment schedule	Number of replans
**180**	23	68	female	AC	distal	3	2	IVA	nCRT	0
**181**	23	75	male	AC	distal	3	1	III	nCRT	0
**182**	23	50	male	AC	distal	3	0	III	nCRT	0
**183**	23	61	male	SCC	distal	3	0	II	nCRT	0
**184**	23	68	male	AC	distal	3	0	III	nCRT	0
**185**	23	73	male	SCC	mid	3	0	II	nCRT	0
**186**	28	75	male	AC	distal	3	2	IVA	dCRT	0
**187**	23	84	male	AC	distal	3	2	IVA	nCRT	0
**188**	23	73	male	AC	distal	2	1	III	nCRT	0
**189**	23	78	male	AC	distal	3	0	III	nCRT	0
**190**	23	54	female	AC	distal	3	1	III	nCRT	0
**191**	23	66	female	AC	distal	3	2	IVA	nCRT	0
**192**	28	78	female	SCC	distal	2	0	II	dCRT	0
